# Diet and Mental Health Relationships in Caribbean Populations: A Scoping Review and Evidence Gap Map

**DOI:** 10.3390/nu18010058

**Published:** 2025-12-23

**Authors:** Catherine R. Brown, Emily Haynes, Khadija Patel, Christina Howitt, Michael Campbell, Madhuvanti Murphy

**Affiliations:** 1 George Alleyne Chronic Disease Centre, University of the West Indies, Jemmotts Lane, St. Michael BB1111, Barbados; khadija.patel@uwi.edu (K.P.); madhuvanti.murphy@uwi.edu (M.M.); 2 European Centre for Environment and Human Health, University of Exeter Medical School, Truro TR1 3HD, UK; 3 The Faculty of Medical Sciences, University of the West Indies, Cave Hill Campus, St. Michael BB11000, Barbados; michael.campbell@uwi.edu

**Keywords:** review, diet, nutrition, food security, mental health, depression, anxiety, Caribbean, SIDS, culture

## Abstract

**Background/Objectives**: Most research linking diet and mental health outcomes is from high-income countries, limiting insight into how these relationships manifest in culturally diverse, vulnerable contexts, such as the Caribbean. This scoping review aims to map existing research on the relationship between aspects of diet and mental health within Caribbean populations, to identify evidence gaps and guide future research. **Methods**: Eleven databases were searched for studies published between 2000 and 2024 in 33 Caribbean countries which assessed the relationship between diet and mental health outcomes. Duplicate screening and extraction were conducted using Redcap software, and a narrative synthesis and evidence gap map were created. The original protocol was registered with Open Science Framework. **Results**: Forty-four records were included, nine of which focused on eating disorders (examined separately). Most were cross-sectional studies of the general population, with few experimental and qualitative studies. Surveys were the most frequently applied data collection tool, often without mention of local adaptation or validation. Most records examined food security and depression as their ‘diet’ and ‘mental health’ variables, respectively. Frequently explored relationships included autism and seafood intake and fruit and vegetable intake, while depression and food security was the most widely examined relationship across studies. **Conclusions**: Caribbean research on diet–mental health relationships is growing though it is limited in scope, design, and cultural validity. Strengthening this evidence base requires studies whose primary aim is in nutritional psychiatry, using culturally relevant tools, and an expansion of study designs that incorporate Caribbean food systems and sociocultural contexts surrounding diet and mental health.

## 1. Introduction

Mental health disorders affect over 970 million people worldwide, making them the leading cause of years lived with disability (one in six) and a significant source of societal and economic strain [1]. Reduced economic productivity and direct costs of care due to mental health are expected to cost the world economy USD 6 trillion by 2030 [1].

Determinants of mental health are wide-reaching, yet the impact of diet on mental health outcomes is gaining increased attention [2,3]. Evidence suggests poor diet quality as a modifiable risk factor for mental health conditions, and existing research has investigated its potential impact in two main ways: the impact of specific nutrients (e.g., Vitamin D, omega-3 fatty acids) and the impact of broader dietary patterns (e.g., ultra-processed food consumption, Mediterranean diet) [1,4,5]. For instance, a 2022 review reports the significance of micronutrients and antioxidants in the development of some psychiatric disorders such as Parkinson’s disease, schizophrenia, and depression [6]. Diets high in ultra-processed foods have been associated with systemic (including neural) inflammation and increased risk for depression [7,8]. Research has also highlighted benefits of plant-based diets on symptomatology of depression and dementia [9,10,11]. Likewise, the ketogenic diet has garnered attention for its potential for improving the symptomatology of dementia, bipolar disorder, schizoaffective disorder, and unipolar depression/anxiety [12,13,14]. Further, the ketogenic diet was added to the UK National Institute for Health and Care Excellence Clinical Guidelines for Epilepsies for drug-resistant paediatric epilepsy [15]. A growing number of reviews explore the relationship between diet and mental health—some focusing on specific diets or food groups (e.g., plant-based or ketogenic diets, fruits and vegetables) and/or mental health conditions (e.g., depression, anxiety) [14,16,17,18,19], others taking a broader focus on these variables [20,21,22]. This growing research has led to the conceptualisation of ‘nutritional psychiatry’ as its own field of work [23].

Despite a growing evidence base, gaps remain in examining diverse cultural, dietary, and environmental contexts. Most studies are conducted in high-income, Western countries such as the United States and Europe, limiting generalisability of findings to low-and-middle-income countries (LMIC) and other under-represented countries such as Small Island Developing States (SIDS). Despite most of the world’s population living in LMICs, less than a quarter of the diet–mental health evidence and only 10–20% of experimental studies on the topic are conducted in these populations [24]. SIDS are also underrepresented in research overall, given their smaller populations and capacities [25]. This evidence gap is significant considering that socioeconomic factors, cultural dietary practices, and differing mental health beliefs may play a role in the exploration of diet–mental health relationships [26,27]. This gap is further accentuated by mutual geographic/climatic, environmental, and economic vulnerabilities of these regions, which disproportionately impact their food systems and contribute to high levels of food insecurity [28,29]. Without evidence from a wider range of settings such as these, global dietary recommendations in support of mental health remain limited in their generalisability.

The Caribbean region, comprising a mix of high and LMICs and 16 of the 39 SIDS [30], represents a prime setting for expanding diet–mental health research. Coupled with rising food insecurity levels within its fragile food system [31,32,33], the region has undergone a nutrition transition from traditional, nutrient-dense whole foods to a reliance on imported, low-nutrient, processed foods [34,35]. Further, Caribbean diets consistently fall below recommended targets for key food groups such as fruits, vegetables, legumes, nuts, and whole grains [36]. Increasing reliance on poor quality foods contributes to a rise in metabolic risk factors including elevated blood pressure, blood glucose, blood lipids, and obesity levels and a growing burden of non-communicable diseases (NCDs), such as diabetes, cardiovascular disease, and cancer [37,38]. These conditions not only account for 75% of regional mortality, but also are frequently comorbid with poor mental health [1]. This physical–mental health syndemic is further fuelled by the region’s significant vulnerability to climate change impacts, global economic shifts, and its own unique cultural histories which can shape both food systems and psychosocial stressors [32].

Adding to this backdrop is the growing mental health burden in the Caribbean. While Caribbean-wide data is limited and largely model-based, Global Burden of Disease data indicate that mental health disorders contribute to 5% of DALYs and 16% of YLD in 2019 in the Caribbean [29,39]. This burden is expected to rise at least until 2050, particularly for dementia [40]. Of note, Guyana and Suriname rank 4th and 7th, respectively, among global suicide rates, with 24.8 and 22.3 deaths per 100,000 in 2021 [41]. The World Health Organization’s (WHO) Comprehensive Mental Health Action Plan 2013–2030 stresses the global need for stronger leadership and governance, comprehensive and integrated services, prevention and promotion strategies, and information systems [42]. Given the growing syndemic and comorbidity of physical NCDs and mental health conditions, regional calls to action have been made to include mental health in the heavy NCD focus of countries’ health agendas, and address their underlying causes and impacts [43,44,45,46]. These calls include stronger leadership, policies, and advocacy in support of mental health and strengthening of the region’s limited mental health data and research in the Caribbean.

Understanding the research landscape of the relationship between diet and mental health in the Caribbean is the first step to exploring the role that modifying the population’s diet could have on the region’s mental health burden. Thus, the aim of this review is to map research conducted in the Caribbean that examine the relationship between diet and mental health outcomes to elucidate research gaps and focus future Caribbean-based research on this topic.

## 2. Methods

This review follows methodological guidance for the conduct of scoping reviews and the Preferred Reporting Items for Systematic reviews and Meta-Analyses extension for Scoping Reviews (PRISMA-ScR) [47,48]. The original protocol was registered with Open Science Framework (https://doi.org/10.17605/OSF.IO/TKJP5).

### 2.1. Definitions of Variables of Interest

The term ‘diet’ for this review refers to any dietary intake, including nutrient intake, dietary patterns, and dietary quality (inclusive of food security). The term ‘mental health’ is broadly defined by the WHO to include mental disorders, psychosocial disabilities, and other mental states associated with significant distress, impairment in functioning, or risk of self-harm [49]. Further details of these definitions are listed in Table 1. The year 2000 was selected as the starting point for the current study as nutrition research concerning mental health increases substantially after this date [2].

### 2.2. Eligibility

Eligibility criteria are listed in Table 1 below.

**Table 1:** Eligibility criteria for review.

Item	Included	Excluded
Study design	Experimental or observational studies (qualitative and quantitative), case reports, and conference proceedings (where full-text/data was available). Reviews are included at the title/abstract screening stage only to identify potentially eligible studies.	Editorials.
Study setting	Countries/territories in the Caribbean region are included, inclusive of Anguilla, Antigua and Barbuda, Aruba/Bonaire/Curacao, The Bahamas, Barbados, St. Bart’s, Belize, Cayman Islands, Cuba, Dominica, Dominican Republic, St. Eustatius, French Guiana, Grenada, Guadeloupe, Guyana, Haiti, Jamaica, St. Kitts and Nevis, St. Lucia, St. Martin, St. Maarten, Martinique, Montserrat, Puerto Rico, St. Vincent and The Grenadines, Saba, Suriname, Trinidad and Tobago, Turks and Caicos, and the Virgin Islands (US and British). This list is based on that of previous Caribbean-based systematic reviews on social determinants of health [50,51,52].	Caribbean diaspora.
Population	Humans of any age residing in the included study settings.	
Intervention	Any intervention; however, studies did not need to implement an intervention to be eligible (i.e., observational designs included).	
Diet variable	Any indicator of ‘diet’, including intake of any nutrient, supplement or food, and assessed using any quantitative dietary assessment method (such as food frequency questionnaires, dietary recall, indices of dietary quality, food insecurity scales) or qualitative method (such as interviews or focus groups).	Variables that are not direct measures of food security or diet intake/quality, including: attitudes to/knowledge of diet, water or alcohol intake, and physical/biological proxies of diet/nutritional status (e.g., stunting, wasting, obesity, biological samples of nutrients).
Mental health variable	Any indicator of ‘mental health’ assessed using relevant symptomatic scales or screening tools (e.g., Patient Health Questionnaire-9 Depression scale), diagnostic criteria (e.g., Diagnostic and Statistical Manual of Mental Disorders) or as experienced subjectively by participants (such as in qualitative studies or other means of self-report). This broad inclusion of symptomatology assessment aligns with recommendations to avoid narrowly focusing on diagnostic criteria which could skew potential differing concepts of mental health [24,53]. Examples of indicators include common disorders such as depression, anxiety, suicidal ideation, as well as less explicit concepts like insomnia. Neurological conditions such as autism, epilepsy, attention deficit and hyperactivity disorders, dementias and Parkinson’s disease are also included. While classified in separate chapters in diagnostic manuals, experts argue the distinction between neurological and psychological is inconsistent with scientific understanding of nervous system disorders with clear mental health components [54].	Proxies for mental health status such as drug or alcohol use.
Additional notes: analysis between diet and mental health variables	Studies reporting on the relationship between at least one diet and one mental health variable. Studies reporting pooled analyses from multiple countries, once at least one Caribbean country is included in the analysis. Eating disorders can be considered a diet indicator and/or a mental health indicator depending on study details. Studies reporting on eating disorders are included but reported separately from diet–mental health relationship records.	Studies examining diet and mental health variables across generations (i.e., maternal mental health and feeding practices of children and vice versa). With eating disorder studies as the exception, studies that do not report on the relationship between at least one diet variable and one mental health variable.
Publication status	Published or unpublished studies released between 1 January 2000 and 11 February 2024 (date of search) in English, Spanish, French, Dutch (i.e., four dominant Caribbean languages).	

### 2.3. Search Strategy

Search terms were conceptualised through examination of those used by other reviews on the topic and discussion with colleagues in the field [2,24,52]. The terms were broad to ensure sensitivity, and an example using PubMed syntax is listed in Supplementary Materials Table S1. Eleven databases were searched: Web of Science (via Clarivate), MEDLINE (via PubMed), EMBASE (via Ovid), SciELO, CINAHL (via EBSCO), CUMED (via WHO Virtual Health Library), LILACS (via WHO Virtual Health Library), PAHO and PAHO IRIS (via WHO Virtual Health Library), MedCarib (via WHO Virtual Health Library), PsycINFO (via Ovid), and Global Health (via Ovid). In addition, researchers scanned reference lists of included full-text records and of systematic reviews retrieved during title/abstract screening.

### 2.4. Record Selection and Data Extraction

Records retrieved from database searching were added to Rayyan reference manager [55]. Authors of inaccessible articles were contacted where contact information was given. Records were reviewed against the eligibility criteria in two steps: (1) Initial screening of titles and abstracts of records → potentially relevant (include) and not relevant (exclude); and (2) Secondary screening of the full-text records identified as potentially relevant in Step 1 → relevant (include) and not relevant (exclude). Key details of eligible records were extracted into REDCap data management software version 15.9.3 [56], including publication information, intervention details (if applicable) and indicators/outcome measures and data collection tools. Screening and data extraction were performed in duplicate by two independent reviewers, and any disagreement or uncertainty was resolved by a third reviewer.

### 2.5. Data Synthesis

The findings are reported narratively as a descriptive summary of publication details and study characteristics including design, settings, intervention, variables, tools applied, and relationships assessed. An evidence gap map was constructed summarising the distribution of existing evidence. Following scoping review methodology, effect measures and study risk of bias were not assessed.

## 3. Results

### 3.1. Summary of Included Records

**Figure 1:**
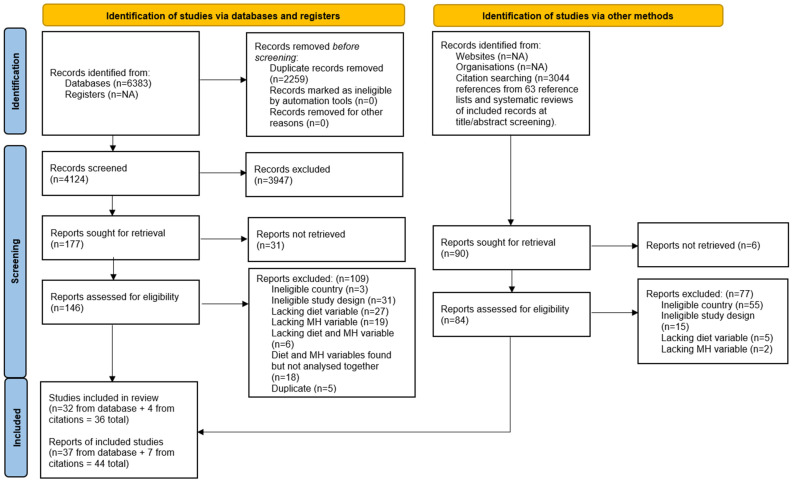
PRISMA flowchart of records [57].

Of the 6318 database search results and 3044 references screened, 44 records from 36 studies were included (See Figure 1). The references for these 44 records are listed (in the same order as the table) in Supplementary Materials Table S2.

Of the 44 records, 35 examined diet and mental health relationships and 9 were classified as ‘eating disorder’ records. Eating disorders are not an aspect of diet but are considered a behavioural condition that affects dietary intake [58]. Although these studies did not fit the primary aim of this scoping review, their relevance to the topic is acknowledged and so they are briefly and separately presented at the end of this Results section. 

**Figure 2:**
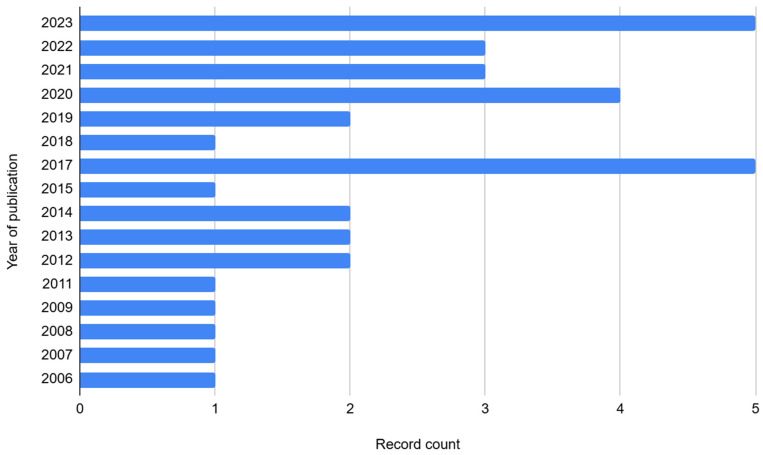
Bar chart of the number of Caribbean diet–mental health relationship records published per year.

The 35 included diet–mental health relationship records were published in 33 peer-reviewed journals, one e-book of studies, and one thesis. Only half were open access. The scope of publications included public health, medical science (broadly), and nutrition (n = 26), with relatively few mental health journals (n = 5). The remaining publications were one unpublished thesis and three environmental journals. Publications spanned from 2006 to 2023, with a gradual increase from one article published per year to five articles per year (See Figure 2).

Studies were conducted in 20 of the 33 Caribbean countries, with most records from Jamaica (n = 12 records), Barbados (n = 7), Dominican Republic, Puerto Rico, and Trinidad (n = 6 each). Others included Cuba and Haiti (n = 4 each); Grenada (n = 3); US Virgin Islands and Bahamas (n = 2 each); and Antigua, Anguilla, Belize, Curacao, Guadeloupe, Martinique, St. Kitts and Nevis, St. Lucia, St. Vincent and the Grenadines, and Suriname (n = 1 each). Overall, 9 of the 35 records reported analyses of pooled data from multiple countries—3 with pooled data from Caribbean countries only [59,60,61] and 6 which also pooled international data [62,63,64,65,66,67].

### 3.2. Study Details

**Figure 3:**
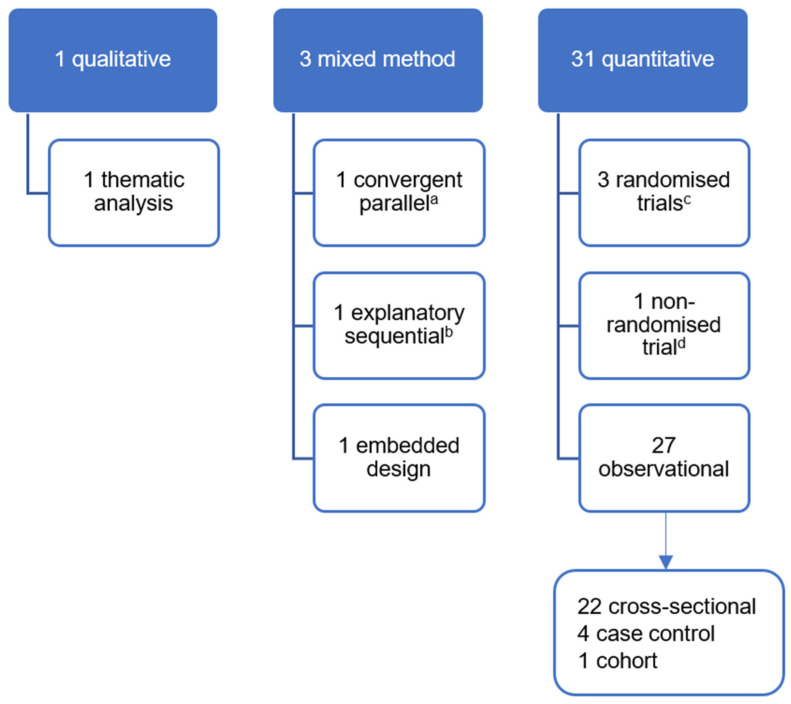
Study designs of records reporting diet–mental health relationships. ^a^—Only qualitative data of this mixed-methods study contributed to this review; ^b^—This mixed-methods study included an uncontrolled before and after non-randomised trial of an intervention; ^c^—All three randomised controlled trials are individually randomised parallel-group trials; ^d^—Case study of one patient.

Study details of the 35 diet–mental health relationship records are listed in Table 2. The sampling frame for most records was the general population/communities (n = 18 records), while others sampled from schools (n = 10), clinics (n = 6), and prison (n = 1). Ten records used child-only samples [67,68,69,70,71,72,73,74,75,76], and some sampled specifically from low-income communities or intentionally recruited participants who were pregnant or reported health issues (e.g., cancer, Parkinson’s disease). Nearly all records used quantitative, observational designs (n = 31) (See Figure 3).

Five records reported on experimental studies [69,79,81,92,93]. One used urban gardens, nutrition, and cooking education to improve food security and subsequently mental and emotional wellbeing among persons living with human immunodeficiency virus in Dominican Republic [81]. Two records from The Jamaican Supplementation and Stimulation Study used milk-based formula supplementation of infants to improve future psychosocial functioning later in life (depression, anxiety, attention deficit, and oppositional behaviour) [92,93]. The final two records implemented a ketogenic diet for (a) adults in Trinidad with various types of cancer to improve their depression and quality of life [79], and (b) a boy in Cuba diagnosed with medication-resistant epilepsy to reduce the frequency and severity of seizure events [69].

Notably, only 10 of 35 records aimed *primarily* to assess the relationship between diet and mental health, versus 20 records for which this was a secondary aim and 5 where it was not an aim at all. Of these 10 studies, 2 sought to examine the impact of a ketogenic diet intervention (mentioned above) [69,79]; 2 sought to examine the association of dietary patterns and perceived stress among students in Puerto Rico [84,88]; 4 examined the association between food insecurity and (a) suicide ideation/plans/attempt among adolescents across the Caribbean [67,75] and (b) mental health status (including anxiety and depression) in vulnerable communities in Haiti [86] and across several Caribbean countries [59]. One record investigated the association between skipping meals and depression and post-traumatic stress disorder symptomatology among students [66], and another sought to examine the association of annonaceae (e.g., sour soup, sugar apple) consumption and cognitive manifestations in atypical forms of parkinsonism and Parkinson’s disease in Guadeloupe and Martinique [82].

Studies from five records examined diet–mental health relationships during or in response to hazards or disasters—two during the COVID pandemic assessing participants in Barbados, Cuba, Dominican Republic, Haiti, and Puerto Rico [64,65] and three during or after hurricanes affecting the Dominican Republic, Puerto Rico, and US Virgin Islands [85,89,91].

### 3.3. Variables and Tools

#### 3.3.1. Indicators of Diet

Of the 35 diet–mental health relationship records, 16 examined food security as the ‘diet’ variable and 19 examined diet intake, quality, or type. Diet intake variables included the intake of macronutrients (e.g., fats, protein) (n = 3 records); supplementation (infant formula) (n = 2); seafood (n = 6); meat (n = 2); fruit and/or vegetable (n = 6); grains (n = 1); meals (n = 2); and snacks (n = 2). Overall diet quality (i.e., diet diversity or food group adequacy) (n = 2) and diet type (i.e., ketogenic diet (n = 2); ‘diabetes mellitus specific’ diet (n = 1); and ‘Western’ diet (n = 1)) (n = 4) were also examined. Most records used food frequency questionnaires to collect dietary data (n = 14); one used 24 h recall and food diary, and another used qualitative interviews. The three remaining experimental records did not explicitly explain their diet assessment procedure [81,92,93].

Food security was most commonly measured using surveys, while two records used qualitative interviews. However, only four records used standardised food security surveys, namely the Household Hunger Scale, Household Food Insecurity Access Scale, Latin American and Caribbean Food Security Scale, and the Food Insecurity Experience Scale Survey Module for Individuals. Others used generalised multi-component surveys, often with only one question assessing food security (n = 10).

Few records explicitly examined processed foods (e.g., cakes, dumplings, soft drinks, Vienna sausages, added sugars) within their diet variables [72,74,84,88,90], and nine records specifically mentioned the production or consumption of local foods. These local foods included annonaceae in Guadeloupe and Martinique [82] and various local fruits and vegetables (e.g., ackee, avocado) in Jamaica [71,72,73,74]. Two examined local food production (a food security intervention) [81] and local food consumption [89] in Dominican Republic. Two others examined individual agricultural assets (a measure of food security) in Haiti [80,86]. All other records assessed diet without specification of local/traditional foods.

#### 3.3.2. Indicators of Mental Health

Depression diagnosis or symptomatology was the most frequently (and widely) examined variable (n = 17 records), followed by perceived stress (n = 8) and anxiety diagnosis/symptomatology (n = 7). Others included suicide ideation/planning (n = 5 records), suicide attempt (n = 2), dementia diagnosis/symptomatology (n = 2), post-traumatic stress disorder symptomatology (n = 2), happiness/enjoyment (n = 2), overall quality of life (includes mental health components) (n = 1), and oppositional behaviour symptomatology (n = 1). Seven records reported on indicators of neurological conditions other than dementia: parkinsonism severity (n = 1), epileptic seizures (n = 1), autism diagnosis (n = 4), and attention deficit behaviours (n = 1). The remaining five records used ambiguous indicators: scores for ‘mental and emotional wellbeing’ (n = 1), ‘mental health symptoms’ (n = 1), ‘negative experience index’ (n = 1), ‘positive experience index’ (n = 2), and having a child at home with a mental disability (n = 1).

A wider variety of tools were used to measure mental health variables than to measure diet variables; 46 unique tools were used for measuring the 16 mental health variables, as shown in Table 3. Among all mental health variables, depression was measured by the widest variety of tools—14 different tools (1 diagnostic test and 13 measures designed to assess symptomatology) across 17 records. On the other hand, the WHO Global School-Based Student Health Survey was used in all five records examining suicidal thoughts and behaviours [67,68,70,75,76]. Some data collection tools assessed multiple different mental health variables in their studies: Geriatric Mental State Exam [94]; Child Vulnerability Survey [60]; WHO Global School-Based Student Health Survey [95]; Behaviour and Activities Checklist [92]; Negative Experience Index [59]; researchers’ own survey; and qualitative interview.

#### 3.3.3. Cultural Adaptation of Tools

Although an array of tools was used to collect data on diet and mental health outcomes, only 10 explicitly noted that the data collection tool was culturally adapted to their Caribbean setting. Three of these were qualitative interview guides are assumed to be designed for defined populations of the studies. Five records culturally adapted their tool for dietary intake data collection by adding culturally relevant food groups or questions and piloting [60,79,88], or researchers used a tool previously created for or adapted to the Caribbean setting (e.g., Latin American and Caribbean Food Security Scale) [61,82]. Regarding mental health tools, four records culturally adapted their tool by adding questions and piloting [60,79] or used a tool previously created for or adapted to the Caribbean setting [80,86].

### 3.4. Relationships Examined

**Figure 4:**
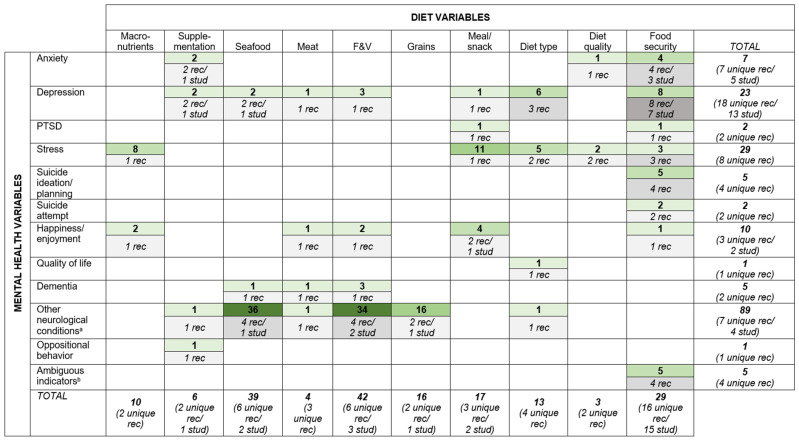
Evidence gap map illustrating the distribution of the 179 diet–mental health relationships across reported diet and mental health variables. Footnote—Numbers in green boxes denote number of relationships examined; numbers in grey boxes denote number of records and studies (where study number is not listed, the records represent their own unique studies); darker colour shading indicates higher numbers of relationships and records. Row and column totals identify the total number of relationships examined for each diet and mental health variable, along with the number of unique records and studies they are found in. ^a^—Other neurological conditions include parkinsonism severity, epileptic seizures, autism diagnosis, and attention deficit behaviours. ^b^—Ambiguous indicators of mental health include ‘mental and emotional wellbeing’, ‘mental health symptoms’, ‘negative experience index’, ‘positive experience index’, and having a child at home with a mental disability.

Below, Table 4 details the relationships examined and tools used in each of the 35 included records. Figure 4 further illustrates the distribution across variables of these 179diet–mental health relationships.

Fruit and vegetable intake (n = 42 relationships), seafood intake (n = 39), and food security (n = 29) were the most *frequently* examined diet variables. However, the majority of the seafood intake variables are from the Jamaican Autism Study examining autism diagnosis [71,72,73,74]. ‘Other neurological conditions’ was the most *frequently* examined mental health variable (n = 89 relationships). However, nearly all are also from the Jamaican Autism Study examining the association between autism diagnosis and the intake of fruits and vegetables, seafood, grains, and meat [71,72,73,74]. Perceived stress (n = 29 relationships) and depression (n = 23) were also frequently reported yet spread across a larger number of unique records/studies (i.e., more *widely* examined). Food security was the most *widely* examined diet variable (n = 29 relationships examined in 16 records from 15 studies), and depression was the most *widely* examined mental health variable (n = 23 relationships examined in 18 records from 13 studies).

With respect to relationships, the most *frequently* examined relationships were seafood intake and other neurological conditions (n = 36 relationships in four records from one study); fruit and vegetable intake and other neurological conditions (n = 34 relationships in four records from two studies); and grain intake and other neurological conditions (n = 16 relationships in two records from one study)—all from the same Jamaican Autism Study. Apart from these, meal/snack intake and perceived stress (n = 11 relationships in one record); macronutrient intake and perceived stress (n = 8 relationships in one record); and food security and depression (n = 8 relationships in eight records from seven studies) were the most *frequently* examined relationships, the latter also being the most *widely* examined relationship of all relationships found.

### 3.5. Eating Disorders

Nine peer-reviewed, mostly cross-sectional records (2002–2011) examined eating disorders; five were open access. They included one pooled regional analysis on dieting behaviours and their correlates across nine countries [96]; five reports of school-based surveys on bulimic disorders, eating attitudes, and body image perceptions in Barbados, Trinidad, and Puerto Rico [97,98,99,100,101]; two medical-record reviews of anorexia and other eating disorder diagnoses in Curacao and Jamaica [102,103]; and one mixed-methods study exploring cultural influences on anorexia diagnoses in Curacao [104]. Together, they assessed incidence, prevalence, correlates, and contextual factors related to eating disorders in adolescents and adults across 12 Caribbean countries.

Except the Jamaica and Curacao studies which were secondary data analyses, the data collection tools included the following:

- To screen for anorexia and/or bulimia: Researchers own short surveys (n = 4 records); Eating Attitudes Test-26 [105] (n = 5); Bulimia Investigatory Test Edinburgh (BITE) [106] (n = 2); Bulimia Test-Revised (BULIT-R) [107] (n = 2); Eating Disorders Inventory (EDI) [108] (n = 1); and the Questionnaire for Eating and Weight problems-revised [109] (n = 1);
- To diagnose bulimia: Diagnostic and Statistical Manual of Mental Disorders, 3rd edition, revised Bulimia Diagnostic Interview [110] (n = 1);
- To screen for any eating disorder: Drive-for-Thinness subscale of the Eating Disorder Inventory-2 [111] (n = 1).

Except for three studies of university students [97,100,101], authors reported a relatively low prevalence of potential eating disorders in the context of the protective factors of a “still traditional” culture [98] of healthy body size preferences and eating habits. Authors referred to the growing influence of Western ideals of thinness/beauty as a contributing factor to eating disorders, while one hypothesised that the influence may not be Western-specific per se but rather related to the process of any society’s complex socio-cultural development [104].

## 4. Discussion

### 4.1. Summary Findings and Evidence Gaps

Thirty-five records examined relationships between diet and mental health in the Caribbean between 2000 and 2024. Despite relatively limited human and infrastructural capacities, an increasing number of studies were conducted in a wide range of Caribbean countries (20 of 33 searched). While this might seem to reflect growing regional interest in the field, the low number of studies that examine this relationship as a *primary* research aim suggests the Caribbean to be at an early, exploratory stage of examination.

With respect to study design, the common use of cross-sectional design limits speculation on the direction of reported associations. Though five interventions were found, the collective strength of evidence is limited due to relatively small sample sizes and lack of control groups or randomisation. The low number of qualitative studies indicates a weak understanding of sociocultural beliefs about the diet–mental health relationship, including what might shape the relationship or its focus in academic research. Overall, studies were mostly epidemiological; there were no biological studies of mechanisms of action (for example, gut or neural inflammation, gut microbiome, or hypothalamic–pituitary–adrenal axis) which could also deepen the regional evidence base on nutritional psychiatry. Finally, with respect to eating disorders, the evidence spans only up to 2011, deeming their findings possibly less relevant considering that Caribbean countries are vulnerable to rapid globalisation and it is argued that prevalence of eating attitudes and disorders may change over time [98].

Although a variety of diet and mental health variables were examined, the Jamaican Autism Study [71,72,73,74] skews the image of a strong Caribbean focus on autism when in fact its high count of relationships comes from this single study. It is unsurprising that perceived stress and depression were the most frequently and widely examined mental health variables, given the ubiquity of stress and its implications for both physical and mental health, and that depression is among the most common mental health disorders globally [112]. Yet two major mental health disorders were not reported - bipolar and schizoaffective disorders. While less prevalent than anxiety or depression, this is a significant evidence gap given their severity of symptoms, comparative difficulty in achieving remission, and the emerging evidence of effective nutritional interventions that may reduce their symptoms and burden of disease [17,113].

The most frequently and widely reported diet variables (fruits and vegetables, seafood and food security) are also unsurprising given the commonly purported protective role of fruits and vegetables in promoting adequate health [18,114], the cultural significance and abundance of seafood in Caribbean diets, and the rise in food insecurity in the region [31]. However, the low number of records examining processed foods and local foods specifically is important considering the Caribbean’s heavy reliance on processed food imports, and the vital role local foods could play in improving food sovereignty, food security and NCD risk profiles in the Caribbean [32,34,35,115]. Indigenous foods of the region have declined in availability since colonialism, but traditional plant-based foods, such as the West Indian cherry, mango, avocado, aloe, cassava, and breadfruit, not only have nutritional benefits, but also their production could improve food sovereignty and climate resilience of small island food systems [116,117].

Though not problematic to see diversity in variables examined across studies, the variation in data collection tools used limits both the comparability between settings and, in some cases, the validity of findings. Variation could obscure observed associations when comparing diverse contexts [59]. For example, the variability of tools used to assess food security is relevant considering that its impact on mental health is not only from biological effects of poor nutrition but also because of the stress caused by being food insecure, with accompanying emotions and behaviours which can be culturally bound [21,118]. Suicide was the only variable examined by the same standardised tool, namely the WHO Global School Based Survey. However, validity of findings is questionable as the survey often used a single yes/no question for assessment (e.g., “During the past 12 months, did you ever seriously consider attempting suicide?”) [68]. This inconsistency in tools is perhaps driven by the relative lack of measures (particularly mental health measures) validated for use in the Caribbean, which raises risk for error in interpretation of both research and clinical findings [119,120]. The emerging field of global mental health emphasises the significance of cultural variation in conceptualisations of mental health, which can influence both the interpretation and measurement of mental health constructs and questions the applicability of standardised assessment tools across diverse settings [26,121]. The same can be said for diet; measuring dietary intake with standard tools such as food frequency questionnaires is challenged by cultural differences in food preferences and varying access to foods, which may not be fully captured. The omission of culturally specific foods can limit the accuracy of dietary assessment, particularly amongst underrepresented groups, potentially contributing to gaps in understanding diet-related health outcomes [27].

### 4.2. Future Research Focus

A more targeted systematic review to follow this scoping exercise is recommended to examine this existing data. Potential areas of focus can include the most widely reported relationships—‘food security and depression’, and ‘food security and suicide ideation/planning/attempt’. Additionally, risk of bias assessments of systematic reviews will elucidate the strength of existing evidence.

Secondly, conducting studies with the *primary* aim of examining the relationship between diet and mental health could deepen understanding of nutritional psychiatry in the region. Study designs that promote enquiry beyond the cross-sectional observational study are warranted. Qualitative and mixed-methods studies can provide insight into why research in this area might be lacking and what cultural beliefs and understandings exist about the relationship. Hoek et al., for example, found sociocultural factors to be associated with the incidence of anorexia nervosa in Curacao, and they call for a deeper understanding to support their explanatory hypotheses on how settings influence the development of eating disorders [102]. Intervention studies would also benefit the regional evidence base of nutritional psychiatry, perhaps focusing on plant-based and ketogenic diets and including more severe mental health conditions given the growing amount of evidence in support of these in other settings [9,12,14]. Interventions promoting fruit and vegetable intake might be particularly relevant for Caribbean populations given existing low intake and potential benefit for both physical and mental health [36]. Further, understanding contextual factors such as cultural food preferences and local food availability and accessibility could complement intervention work. Researchers should also consider examining processed food and local food consumption and use dietary diversity as a proxy for nutrient adequacy, given their evidenced impact on mental health in other countries and considering the Caribbean’s narrowing local foodscape and dependency on (often processed) food imports [117,122]. Likewise, researchers should facilitate ethical inclusion of people with severe mental illness, such as those with bipolar disorder and schizophrenia, to increase their representation in needed research [123]. Though, researchers must acknowledge the threat of Neyman bias arising from the potential of persons with severe mental health disproportionately refusing to participate in or withdrawing from studies, and also the difficulty in recruiting from other hard-to-reach groups (i.e., low-income or rural dwellers). Finally, social desirability bias is also at play in any self-report of diet, where participants might erroneously report healthier foods than those that are truly consumed.

With respect to tools, researchers are encouraged to use culturally appropriate data collection tools or conduct validation studies in Caribbean settings to prevent the misappropriation of WEIRD-developed tools (‘Western, Educated, Industrialised, Rich and Democratic’ contexts) [124]. For instance, the Beck Depression Inventory II has been validated in Jamaican university students [125]; Perceived Stress Scale and Brief Resilient Coping Scale were validated in health professional students across four Caribbean countries [124]; and EQ-5D-5L for self-reported health was validated in adults in Barbados and Jamaicas [126]. Yet caution must be taken in applying these tools across all ages and countries given the specificity of study samples. Also, any cultural adaptation to standardised tools must also appreciate the ‘tailor locally, retain globally’ principle in measurement tool adaptation. While culturally adapting standardised tools is essential, the process must be balanced to preserve enough of the original structure to allow for meaningful cross-country comparisons [127]. A recent example is the adaptation and pilot of the INTAKE24 dietary intake data collection tool, which was originally developed for the United Kingdom context. Researchers from the Global Community Food and Health Project adapted the diet recall tool for application in St. Vincent and the Grenadines, adding Caribbean dishes and local foods to ensure cultural applicability and validity (ongoing study; manuscript in review) [128]. Food frequency questionnaires can also be adapted to include context-specific foods and use more branching logic with open-answered questions to capture these foods in a systematic way [27].

Finally, researchers are encouraged to publish in more open-source platforms and mental health-focused publications to promote further awareness and advocacy in mental health arenas where, perhaps, knowledge on the potential impact of diet on mental health may be less acknowledged.

### 4.3. Strengths and Limitations

To our knowledge, this scoping review is the first to attempt to map research on diet and mental health relationships in the Caribbean. It provides a broad snapshot of existing research to guide focused systematic reviews and primary studies on the topic. Its broad search strategy encompassing eleven databases and the inclusion of review articles during screening maximised sensitivity to capture as many relevant studies as possible. However, as expected within scoping review work, researchers were challenged by deciding on eligibility criteria for the two variables. While definitions of mental health were kept broad, researchers were forced to draw a line between what constitutes ‘mental health’ versus ‘overall brain health’, especially considering the physiological connection between physical and mental health and the shared purported pathophysiology of diet’s impact on both, through oxidative stress and systemic inflammation, for example [3]. It is acknowledged that broader definitions of variables might identify further studies.

## 5. Conclusions

Research examining diet and mental health relationships is steadily increasing in Caribbean contexts. However, research is limited in study design (mostly cross-sectional); is thinly spread across most variables; and often uses data collection tools that may not be culturally appropriate. Targeted systematic reviews of the identified diet and mental health variables are required to better understand the extent of existing evidence in the region. Experimental and qualitative studies that *primarily* aim to explore the impact of diet on mental health outcomes and related beliefs of such, using culturally appropriate, data collection tools, are warranted. Diet and mental health are cultural phenomena of populations and future examinations should be performed with a cultural lens to elucidate more on if and how culture interacts with findings.

## Figures and Tables

**Table 2:** Study design details of 35 included diet–mental health relationship records [59,60,61,62,63,64,65,66,67,68,69,70,71,72,73,74,75,76,77,78,79,80,81,82,83,84,85,86,87,88,89,90,91,92,93].

First Author, Year of Publication (Study Name, Where Applicable)	Country	Sampling Frame	Age (Years)	Sample Size	Study Design	Aim of Study	Explanatory Theory or Model
Albanese, 2009 (10/66 Study) ^a^ [77]	Cuba, Dominican Republic (plus 5 other countries)	Community (urban areas)	65+	Cuba = 2934; DR = 1999	Quantitative: cross-sectional	Overall study: To create a pubic database of dementia prevalence/incidence and associations across many variables, and to validate its 10/66 survey. Report: (1) To describe fish and meat intakes and their relation to the health and sociodemographic characteristics of older people across countries; (2) To test the hypotheses that dietary fish is inversely associated and dietary meat is directly associated with prevalent dementia; (3) To test the consistency of the country-specific hypothesised inverse association of fish and dementia with control for the relevant confounders and after having disaggregated the potential concomitant opposing effect of meat consumption.	Neuoroprotection of omega-3 fatty acids.
Albanese, 2012 (10/66 Study) ^a^ [78]	Cuba, Dominican Republic (plus 5 other countries)	Community (urban areas)	65+	Cuba = 2944; DR = 2011	Quantitative: cross-sectional	Overall study: To create a public database of dementia prevalence/incidence and associations across many variables, and to validate its 10/66 survey. Report: To test the strength of the relationship of fish consumption with prevalent depression in large representative samples of community-dwelling older people.	Neuoroprotection of omega-3 fatty acids.
Augustus, 2020 ^f^ [79]	Trinidad and Tobago	Clinic (cancer patients)	Only means stated: intvn group—49.80 ± 6.72; control group—51.80 ± 4.18	40 total; 20 intvn; 20 control (16 intvn in analysis)	Quantitative randomised trial: individually randomised parallel-group trial	(1) To examine the use of the KD and adaptation period in association with the management of cancer; (2) To understand the effects of the KD on the functional, nutritional, psychosocial, and overall QoL among cancer patients.	Ketosis to reduce glucose metabolism of tumour cells “Warburg effect” which could improve quality of life.
Benites-Zapata, 2021 [65]	Dominican Republic, Haiti, Puerto Rico (plus 17 other Latin American countries)	General population (Facebook users)	18+	1,324,272 (all countries; individual countries not listed)	Quantitative: cross-sectional	To know the prevalence and factors associated with food insecurity in LAC countries during the early stage of quarantine due to COVID-19.	COVID-19 pandemic has caused a social, economic, and health impact.
Brewis, 2020 (Haiti Justice Sector Strengthening Project) ^b^ [80]	Haiti	Community (vulnerable communities)	Not stated; “head of household”, likely 18+	Survey—4055; Qual—95 FGs and interviews	Mixed methods (embedded) using cross-sectional survey, interviews, focus groups	To use comparison across sites and across diverse data forms to consider how syndemics manifests differently across communities that share the same basic cultural understandings and broad structural limitations, i.e., how they localise—using applying two datasets collected using different epistemological frameworks.	Syndemic localisation: hunger interacts with other factors (crime, discrimination), impacting mental health (among other outcomes).
Celeste-Villalvir, 2023 (ProMeSA) [81]	Dominican Republic	Clinic (patients living with HIV)	18+	45 (intervention only; no control in analysis)	Mixed methods (explanatory sequential) using quantitative non-randomised trial (uncontrolled before and after study) and interviews	To explore feasibility, acceptability and implementation challenges to inform a larger intervention trial as well as other interventions designed to address food insecurity in low-resource settings.	Bilateral links between food insecurity and HIV/AIDS: food insecurity → increase anxiety, depression and internalised stigma → reduce treatment adherence. Intervention could reduce food insecurity, thereby improving medication adherence and HIV outcomes.
Cleret de Langavant, 2022 ^f^ [82]	Guadeloupe, Martinique	Clinic (patients with degenerative parkinsonism)	30 to 90	180 (from cohort of 350)	Quantitative: cross-sectional	To investigate the link between annonaceae consumption and clinical manifestations not only in atypical forms of parkinsonism but also in Parkinson’s disease.	Possible neurotoxicity of acetogenins in annonaceae.
DaSantos, 2021 [83]	Barbados	Clinic (patients with type 2 diabetes)	20 to 80	509	Quantitative: cross-sectional	To explore the relationship of self-care adherence, and the affective emotional disorders of diabetes distress and depression among Barbadian adults with type 2 diabetes.	Biopsychosocial model: The presence of psychological issues can often result in poor clinical outcomes because of its possible effect on adherence to medication and self-care regimes (e.g., diet).
Ertl, 2022 [64]	Barbados, Cuba (plus 57 other countries)	General population (online survey)	18 to 94	6882 (all countries; individual countries not listed)	Quantitative: cross-sectional	To understand how degree of exposure to the COVID-19 pandemic, lifestyle changes due to quarantine, and associated secondary sequelae were linked with trauma-related distress among adults in the general population.	Disaster trauma theory: variety of factors contribute (e.g., diet) to post-disaster psychopathology, such as trauma-related distress.
Fabián, 2013 ^f^ [84]	Puerto Rico	School (first- and second-year medical students)	21+	275	Quantitative: cross-sectional	To describe the dietary patterns of students and to describe, as well, the association of dietary patterns with socio-demographic characteristics and academic stress.	University environment can change student diet patterns which can in turn negatively impact health of student.
Jeffers, 2022 [85]	US Virgin Islands	General population (pregnant women)	18 to 44	18	Qualitative (interviews)	To describe the pregnancy and birth experiences of women in the USVI following Hurricanes Irma and Maria.	(1) UNICEF Conceptual Framework for Maternal and Neonatal Morbidity and Mortality and (2) Bronfenbrenner’s ecological systems theory: risk and resilience can arise from a variety of interrelated settings and can influence maternal and neonatal health following hurricane impact (e.g., on infrastructure including food systems).
Jones, 2017 ^f^ (2014 Gallup World Poll) [59]	Entire region (countries not stated)	General population	15+	190,348 (LAC = 19,184)	Quantitative: cross-sectional	To determine the association and assess heterogeneity by age and sex of individual-level food insecurity with mental health status across all global regions.	(1) Food insecurity → uncertainty → stress response → depression. (2) Food insecurity → socially unacceptable ways of acquiring food (i.e., food aid) → shame/guilt etc → depression. (3) Food insecurity → magnifies socioeconomic disparities → lower mental wellbeing.
Koyanagi, 2019 ^f^ [75]	Antigua and Barbuda, Bahamas, Belize, Curacao, Jamaica, St. Kitts and Nevis, Trinidad and Tobago	School (high school students)	13 to 15	179,771 (Antigua—1266; Bahamas—1357; Belize—2112; Jamaica—1623; St Kitts—1740; Suriname—1698; Trinidad—2 811)	Quantitative: cross-sectional	To assess the association between food insecurity and suicide attempts, and to examine whether the association is similar across countries (including country-income levels).	Poor nutrition, stress, shame in food insecurity (in children themselves and in parents which affects parenting) may lead to increased risk for mental health problems.
Kwangu, 2017 [68]	Bahamas	School (secondary school students)	13 to 17	1357	Quantitative: cross-sectional	To estimate the prevalence of suicidal ideation and determine its associated factors.	Shame gap from poverty with high expectations versus reality can lead to suicide contemplation.
Lachaud, 2019 (Haiti Justice Sector Strengthening Project) ^b,f^ [86]	Haiti	Community (vulnerable communities)	18+	4055	Quantitative: cross-sectional	To consider how lack of agricultural assets, as a specific dimension of poverty, is associated with common mental disorder symptoms.	Syndemic relationship: worse socioeconomic predicts worse mental health outcomes, which can in turn increase economic risk.
LaMonaca, 2018 [87]	Haiti	Prisons (prisoners)	Not stated; likely 18+.	290	Quantitative: cross-sectional	To characterise the physical and mental health of prisoners in rural Haiti and to examine the effects of having visitors, linked to increased access to food, and length of detention on health status.	Prisoners typically have higher rates of illness and nutritional inadequacies.
López-Cepero, 2021 (Puerto Rico Assessment of Diet, Lifestyle, and Diseases) ^f^ [88]	Puerto Rico	Clinic	30 to 75	380	Quantitative: cross-sectional	To explore the association between perceived stress and intake of macronutrients and diet quality among adults	Puerto Ricans experience both suboptimal dietary intake and high levels of psychosocial stress.
Marcos Plasencia, 2007 ^f^ [69]	Cuba	Clinic (case study child)	2	1	Quantitative non-randomised trial: uncontrolled before and after study	To examine the effects of a ketogenic diet on a 2-year-old suffering from West Syndrome with seizures not amenable to anti-epileptic drugs.	Ketosis assists in ameliorating epileptic seizures due to their anti-convulsant properties.
Martinez-Brockman, 2023 (Eastern Caribbean Health Outcomes Research Network cohort study) [61]	Barbados, Puerto Rico, Trinidad and Tobago, US Virgin Islands	General population	40+	1939	Quantitative: cohort	Overall study: To measure the prevalence and incidence of diabetes, cancer, and heart disease as well as known and potential risk factors including food insecurity. Report: To examine the demographic, psychosocial, behavioural, and environmental risk factors associated with household food insecurity among adults 40+ years of age in the ECHORN cohort.	Evidence of Caribbean having high rates of food insecurity, which has been associated with chronic disease (e.g., diabetes, hypertension etc).
Mulenga, 2017 [70]	Trinidad and Tobago	School (secondary school students)	13 to 17	2811	Quantitative: cross-sectional	To determine correlates for suicidal ideation including its prevalence among in-school adolescents in Trinidad and Tobago.	None stated
Peltzer, 2015 ^c^ [63]	Barbados, Grenada, Jamaica (plus 23 other countries)	School (university students)	Only mean stated: 20.8 +/−2.8	17,789 (Barbados 370; Grenada 418; Jamaica 681)	Quantitative: cross-sectional	To assess the prevalence of fruits and vegetable consumption and associated factors among university students from 26 low-, middle-, and high-income countries.	Evidence of psychosocial factors related to low prevalence of fruit and vegetable consumption.
Peltzer, 2017 ^c^ [62]	Barbados, Grenada, Jamaica (plus 21 other countries)	School (university students)	17 to 30	17,789 (Barbados 505; Grenada 420; Jamaica 742)	Quantitative: cross-sectional	To examine health behaviours and happiness and associated factors in low-, middle-, and high-income countries.	Evidence of association between happiness and range of health behaviours, sociodemographic and social factors.
Pengpid, 2020 ^c,f^ [66]	Barbados, Grenada, Jamaica	School (university students)	17 to 30	21,972 (all countries; individual countries not listed)	Quantitative: cross-sectional	To investigate the associations between skipping breakfast and various health risk behaviours and mental health in university students in 28 countries.	Skipping breakfast is a common practice among university students and has been associated with health compromising behaviours and mental health.
Pengpid, 2023 ^f^ [67]	Anguilla, Dominican Republic, Jamaica, Suriname, Trinidad and Tobago	School (secondary school students)	11 to 18	9956	Quantitative: cross-sectional	To assess associations between food insecurity and multiple psychological and behavioural problems among adolescents in five Caribbean countries.	Evidence of association between food insecurity and mental health outcomes and increasing food insecurity in Caribbean.
Racine, 2008 (Eastern Caribbean Child Vulnerability Study) [60]	Barbados, St. Lucia, St. Vincent and the Grenadines	General population (households with children)	Not stated; likely 18+	2344	Quantitative: cross-sectional	To examine the relationship between food insecurity and child wellbeing indicators.	Evidence of association between food insecurity and psychosocial outcomes and mental wellbeing.
Rahbar, 2012 (Jamaican Autism study) ^d^ [71]	Jamaica	General population (children with autism)	2 to 8	130 (65 cases; 65 controls)	Quantitative: case–control	Overall study: To investigate possible associations between autism spectrum disorder (ASD) and postnatal environmental exposure to five heavy metals of interest: lead, mercury, arsenic, cadmium, and manganese. Record: To examine whether environmental exposures to several heavy metals, including arsenic, have a role in the onset of an ASD.	Arsenic neurotoxicity—through contaminated foods—could contribute to autism spectrum disorder.
Rahbar, 2013 (Jamaican Autism study) ^d^ [73]	Jamaica	General population (children with autism)	2 to 8	130 (65 cases; 65 controls)	Quantitative: case–control	Overall study: To investigate possible associations between autism spectrum disorder (ASD) and postnatal environmental exposure to five heavy metals of interest: lead, mercury, arsenic, cadmium, and manganese. Record: (1) To investigate the association between blood mercury concentrations in children and ASDs; (2) To investigate the role of seafood consumption in relation to blood mercury concentrations in Jamaican children.	Mercury neurotoxicity—through contaminated foods—could contribute to autism spectrum disorder.
Rahbar, 2014 (Jamaican Autism study) ^d^ [74]	Jamaica	General population (children with autism)	2 to 8	220 (110 cases; 110 controls)	Quantitative: case–control	Overall study: To investigate possible associations between autism spectrum disorder (ASD) and postnatal environmental exposure to five heavy metals of interest: lead, mercury, arsenic, cadmium, and manganese. Record: (1) To investigate whether there is an association between blood cadmium concentrations and ASD in children. (2) To estimate blood cadmium concentrations in controls and identify factors associated with blood cadmium concentrations, with a particular focus on the food consumed by these children.	Cadmium neurotoxicity—through contaminated foods—could contribute to autism spectrum disorder.
Rahbar, 2014 (Jamaican Autism study) ^d^ [72]	Jamaica	General population (children with autism)	2 to 8	218 (109 cases; 109 controls)	Quantitative: case–control	Overall study: To investigate possible associations between autism spectrum disorder (ASD) and postnatal environmental exposure to five heavy metals of interest: lead, mercury, arsenic, cadmium, and manganese. Record: (1) To investigate the possible association between blood manganese concentration and ASD status. (2) To identify factors associated with blood Manganese concentration in typically developing Jamaican children.	Manganese neurotoxicity—through contaminated foods—could contribute to autism spectrum disorder.
Rivera, 2023 [89]	Dominican Republic	General population	18+	19 qualitative (relevant data) + 74 quantitative (not relevant data)	Mixed methods (convergent parallel) using cross-sectional survey (data not relevant) and interviews (relevant data)	(1) Qualitative: To explore Dominicano’s experience with using Dominican folk knowledge and practices as self-care; (2) Quantitative: To determine if using Dominican folk knowledge and self-care practices during crisis in the DR improve wellbeing and resilience.	(1) Orem’s Self-Care Deficit Theory, (2) Seligman’s PERMA Theory of Well-Being, (3) Resilience Theory: self-care can affect wellbeing and resilience.
Rocke, 2020 [90]	Trinidad and Tobago	School (university students)	16 to 40	800	Quantitative: cross-sectional	To determine the prevalence of moderate to severe depression and to identify predictors for depression and high levels of stress among university students attending a Caribbean university.	None stated.
Simeone, 2023 [91]	Puerto Rico	General population (women with a recent live birth)	19+	517	Quantitative: cross-sectional	To understand hurricane-related experiences, maternal health concerns, and the impact of hurricane experiences on postpartum depressive symptoms.	None stated.
Siziya, 2017 [76]	Jamaica	School (secondary school students)	Not stated—grades 7–12 (approximately 12–18-year-olds)	1623	Quantitative: cross-sectional	To determine prevalence of suicidal ideation as well as factors associated with suicidal ideation among students in grades 7–12 in Jamaica.	None stated.
Walker, 2006 (The Jamaican supplementation and stimulation study) ^e^ [92]	Jamaica	Community (stunted children from poor neighbourhoods)	Trial age—9–24 months; follow-up age at this study—17/18 years	Original trial 22 years prior—129; At follow-up—103 (stimulation—21; supplementation—28; stimulation and supplementation—27; control—27)	Quantitative randomised trial: individually randomised parallel-group trial	To determine whether previous developmental benefits of stimulation and supplementation were sustained to adolescence (17/18 years old) and to examine possible effects on educational attainment, general knowledge, social and sexual relationships, social inhibition, and antisocial behaviour.	Experiences in early childhood can have long-term effects on brain function and cognitive and psychosocial functioning.
Walker, 2011 (The Jamaican supplementation and stimulation study) ^e^ [93]	Jamaica	Community (stunted children from poor neighbourhoods)	Trial age—9–24 months; follow-up age at this study—22 years	Original trial 22 years prior—129; At follow-up—105 (stimulation—24; supplementation—26; stimulation and supplementation—29; control—26)	Quantitative randomised trial: individually randomised parallel-group trial	To determine whether previous developmental benefits of stimulation and supplementation were sustained to adulthood (22 years old) and to examine possible effects on educational attainment, general knowledge, social and sexual relationships, social inhibition, and antisocial behaviour.	Experiences in early childhood can have long-term effects on brain function and cognitive and psychosocial functioning.

Footnote: ^a^—Two records from the same study (10/66 Study); ^b^—Two records from the same study (Haiti Justice Sector Strengthening Project); ^c^—Three records from the same study (no study name); ^d^—Four records from the same study (Jamaican Autism Study); ^e^—Two records from the same study (Jamaican Supplementation and Stimulation Study); ^f^—Primary aim of record was to assess relationship between diet and mental health.

**Table 3:** Mental health variables examined and data collection tools used.

Mental Health Variable Examined (n = Number of Records)	Number of Tools Used	Name of Tools Used (n = Number of Records)
Depression diagnosis or symptoms (n = 17) (2 diagnostic; 14 symptomatology (including 1 for postpartum depression, 1 for sadness); 1 for child in home with depression treatment)	14	Geriatric Mental State Exam (n = 1); International Classification of Diseases −10 (n = 1); EUROpean Depression scale (n = 1); Patient Health Questionnaire-9 (PHQ-9) (n = 2); Patient Health Questionnaire (PHQ-2) (n = 1); Zanmi Lasante Depression Symptom Inventory (ZLDSI) (n = 1); Hopkins Symptom Checklist (HSCL) (n = 1); Centre for Epidemiologic Studies Depression Scale (CES-D 10 items) (n = 2); Child Vulnerability Survey (n = 1); 21-item Beck Depression Inventory (BDI) (n = 2); Pregnancy Risk Assessment Monitoring System (PRAMS) Disaster supplement(n = 1); Short Mood and Feelings Questionnaire (n = 2); Negative Experience Index (n = 1); researchers’ own survey (n = 1 one Q only)
Dementia diagnosis or symptoms (n = 2) (2 diagnostic; 7 symptomatology)	9	Geriatric Mental State Exam (n = 1); Community Screening Instrument for Dementia (CSI’D’) COGSCORE (part of cognitive battery test) (n = 1); Modified CERAD 10 word list learning task with delayed recall (part of cognitive battery test) (n = 1); Informant interview using CSI’D’ RELSCORE (n = 1); Informant interview using the History and Aetiology Schedule—Dementia Diagnosis and Subtype (HAS-DDS) (n = 1); NEUROEX neurological assessment (n = 1); Neuropsychiatric Inventory (NPI-Q) and Informant Questionnaire (for behavioural and psychological symptoms) (n = 1); Diagnostic and Statistical Manual of Mental Disorders-IV (n = 1); Mattis Dementia Rating Scale (MDRS) (n = 1 for parkinsonism)
Neurological conditions other than dementia (n = 7) (1 parkinsonism severity, 1 epilepsy seizures, 4 autism diagnosis/symptomatology, 1 attention deficit symptomatology)	7	Parkinsonism severity: Hoehn and Yahr scale and Schwab (n = 1), England activities of daily living scale (n = 1); Epileptic seizures: Physician or parent observation (not stated which) (n = 1); Autism diagnosis/symptoms: Autism Diagnostic Observation Schedule (ADOS) (n = 4); Autism Diagnostic Interview-Revised (ADI-R) (n = 4); Lifetime form of the Social Communication Questionnaire (SCQ)(n = 4); Attention Deficit Symptoms: Behaviour and Activities Checklist
Anxiety diagnosis or symptoms (n = 7) (All 7 symptomatology)	6	Beck Anxiety Inventory (BAI) (n = 2); WHO Global School-Based Student Health Survey (n = 1); State-trait Anxiety Inventory (n = 1); Revised Children’s Manifest Anxiety Scale (RCMAS) (n = 1); Researchers’ own survey (n = 1 one Q only); Qualitative interview (n = 1)
Perceived stress = 8 (Including 1 diabetes distress specifically)	5	Diabetes Distress Scale (DDS) (n = 1); Perceived Stress Scale (PSS) (n = 3); Negative Experience Index (n = 1); Researchers’ own survey (n = 1); Qualitative interview (n = 2)
Happiness/enjoyment (n = 2)	2	Subjective Happiness Scale (SHS) (n = 2); Positive Experience Index (n = 1)
PTSD symptoms (n = 2) (Including 1 ‘trauma-related distress’ from COVID specifically)	2	Child-Revised Impact of Events Scale (CRIES-8) (n = 1); Breslau’s 7-item screener (n = 1)
Quality of life (n = 1)	1	European Organisation for Research and Treatment of Cancer current core questionnaire (EORTC QLQ-C30) (n = 1)
Suicide ideation/planning (n = 4)	1	WHO Global School-Based Student Health Survey (n = 4)
Suicide attempt (n = 2)	1	WHO Global School-Based Student Health Survey (n = 2)
Child in home with mental disability (n = 1)	1	Child Vulnerability Survey (n = 1)
Oppositional behaviour symptoms (n = 1)	1	Behaviour and Activities Checklist (n = 1)
‘Mental and emotional wellbeing’ (n = 1)	1	Researchers’ own survey (n = 1, one Q only)
‘Mental health symptoms’ (n = 1)	1	SF-12 Health Survey (SF12) (mental health components of this) (n = 1)
‘Negative experience index’ (n = 1)	1	Negative Experience Index (n = 1)
‘Positive experience index’ (n = 1)	1	Positive Experience Index (n = 1)

**Table 4:** List of relationships examined in each of the 35 diet–mental health relationship records [59,60,61,62,63,64,65,66,67,68,69,70,71,72,73,74,75,76,77,78,79,80,81,82,83,84,85,86,87,88,89,90,91,92,93].

First Author, Year of Publication (Study Name, Where Applicable)	Relationships Assessed	Tools to Measure Diet Outcomes	Tools to Measure Mental Health Outcomes
Albanese, 2009 (10/66 Study) ^a^ [77]	(1) Fish intake and depression diagnosis; (2) Meat intake and depression diagnosis; (3) Fish intake and dementia diagnosis; (2) Meat intake and dementia diagnosis	Survey—food frequency questionnaire	Depression: (Geriatric Mental State Exam) Dementia: (1) Geriatric Mental State Exam; (2) A cognitive test battery comprising (a) the Community Screening Instrument for Dementia (CSI’D’) COGSCORE (incorporating the CERAD animal naming verbal fluency task) and (b) the modified CERAD 10 word list learning task with delayed recall; (3) Informant interview using CSI’D’ RELSCORE; (4) Informant interview using the History and Aetiology Schedule—Dementia Diagnosis and Subtype (HAS-DDS), a modification of the earlier HAS; (5) NEUROEX neurological assessment; (6) Neuropsychiatric Inventory (NPI-Q) and Informant Questionnaire (for behavioural and psychological symptoms); (7) DSM IV for final diagnosis (based on log. Reg. equation developed in their pilot study OR direct application of DSM IV)
Albanese, 2012 (10/66 Study) ^a^ [78]	Fish intake and depression diagnosis	Survey—food frequency questionnaire	(1) International Classification of Diseases −10; (2) EUROpean Depression scale
Augustus, 2020 ^f^ [79]	(1) Ketogenic diet and depressive symptoms; (2) Ketogenic diet and quality of life	(1) 24 h recall (using Ketogenic Nutrition Intervention for Cancer Survey); (2) food diary	Depression: Patient Health Questionnaire-9 (PHQ-9); Quality of life: European Organisation for Research and Treatment of Cancer current core questionnaire (EORTC QLQ-C30)
Benites-Zapata, 2021 [65]	(1) Food insecurity and depressive symptoms; (2) Food insecurity and anxiety symptoms	Survey—one question in researchers’ survey	Researchers’ own survey via Facebook, with two questions adapted from the Kessler Psychological Distress Scale (for both—one adapted question to measure depression and another adapted question to measure anxiety)
Brewis, 2020 (Haiti Justice Sector Strengthening Project) ^b^ [80]	(1) Food insecurity and depressive symptoms; (2) Food insecurity and anxiety symptoms	Survey—Household Hunger Scale	Depression: (1) Beck Depression Inventory-II Anxiety: (1) Beck Anxiety Inventory (BAI)
Celeste-Villalvir, 2023 (ProMeSA) [81]	(1) Participation in food security intervention and “mental and emotional wellbeing”	(1) Survey (process evaluation); (2) Qualitative interviews	Researchers’ own survey (process evaluation)—one question
Cleret de Langavant, 2022 ^f^ [82]	(1) Annonacea fruits/juices intake and cognitive performance; (2) Annonacea tea intake and cognitive performance; (3) Annonacea fruits/juices/tea intake and cognitive performance; (4) Annonacea fruits/juices intake and severe degenerative parkinsonism; (5) Annonacea tea intake and severe degenerative parkinsonism; (6) Annonacea fruits/juices/tea intake and severe degenerative parkinsonism	Survey—food frequency questionnaire	Severity atypical Parkinsonism and Parkinson’s disease: (1) Hoehn and Yahr staging and rating of Schwab and England activities of daily living; Global cognitive efficiency: (2) Mattis Dementia Rating Scale (MDRS)
DaSantos, 2021 [83]	(1) General diet consumption and diabetes distress; (2) Specific diet consumption (diabetes supportive) and diabetes distress; (3) General diet consumption and depressive symptoms; (4) Specific diet consumption (diabetes supportive) and depressive symptoms	Survey—Diabetes Self-care Activities Scale	Depression: (1) Patient Health Questionnaire-9 (PHQ-9); (2) Diabetes Distress Scale (DDS) (for diabetes distress specifically)
Ertl, 2022 [64]	Food insecurity and trauma-related distress from COVID-19	Survey—one question in portion of Epidemic-Pandemic Impacts Inventory (EPII)	Child-Revised Impact of Events Scale (CRIES-8)
Fabián, 2013 ^f^ [84]	(1) Soft drink intake and perceived stress; (2) Chocolate intake and perceived stress; (3) Cookie intake and perceived stress; (4) Nutrition bar intake and perceived stress; (5) Oatmeal cookie intake and perceived stress; (6) Chip intake and perceived stress; (7) Candy intake and perceived stress; (8) Peanut intake and perceived stress; (9) Cake intake and perceived stress; (10) Vienna sausage intake and perceived stress; (11) Nutritional drink intake and perceived stress; (12) Dietary pattern adequacy and perceived stress	(1) Survey—food frequency questionnaire; (2) Modified diet quality index (at analysis stage)	Researchers’ own survey (adapted from another (inaccessible) study with 27 questions on academic stress)
Jeffers, 2022 [85]	Food insecurity and perceived stress	Qualitative interview	Qualitative interview
Jones, 2017 ^f^ (2014 Gallup World Poll) [59]	(1) Food security and negative experience index; (2) Food security and positive experience index; (3) Food security and worry; (4) Food security and sadness; (5) Food security and stress; (6) Food security and smiles/laughs a lot; (7) Food security and enjoyment	Survey—Food Insecurity Experience Scale Survey Module for Individuals (FIES SM-I)	Negative Experiences: (1) Negative Experience Index; Positive Experiences: (1) Positive Experience Index; Sadness: (1) Negative Experience Index; Stress: (1) Negative Experience Index; Enjoyment: (1) Positive Experience Index;
Koyanagi, 2019 ^f^ [75]	Food insecurity and suicide attempt	Survey—one question in Global School-based Student Health Survey	WHO Global School-Based Student Health Survey
Kwangu, 2017 [68]	Food insecurity and suicide ideation	Survey—one question in WHO Global School-Based Student Health Survey	WHO Global School-Based Student Health Survey
Lachaud, 2019 (Haiti Justice Sector Strengthening Project) ^b,f^ [86]	(1) Food insecurity and depressive symptoms; (2) Food insecurity and anxiety symptoms	Survey—Household Food Insecurity Access Scale	Depression: Zanmi Lasante Depression Symptom Inventory (ZLDSI); Anxiety: Beck anxiety inventory (BAI)
LaMonaca, 2018 [87]	(1) Food insecurity and depressive symptoms; (2) Food insecurity and perceived stress; (3) Food insecurity and ‘mental health’ symptoms	Survey—one question in researchers’ survey	Depression: Hopkins Symptom Checklist (HSCL); Stress: Perceived Stress Scale (PSS); ‘Mental health’ components of quality of life: SF-12 Health Survey (SF12)
López-Cepero, 2021 (Puerto Rico Assessment of Diet, Lifestyle, and Diseases) ^f^ [88]	(1) Fibre intake and perceived stress; (2) Starch intake and perceived stress; (3) Added sugar intake and perceived stress; (4) Animal protein intake and perceived stress; (5) Vegetable protein intake and perceived stress; (6) Saturated fatty acid intake and perceived stress; (7) Monounsaturated fatty acid intake and perceived stress; (8) Polyunsaturated fatty acid intake and perceived stress	(1) Food frequency questionnaire; (2) Alternate Healthy Eating Index (at analysis stage)	Perceived Stress Scale (PSS)
Marcos Plasencia, 2007 ^f^ [69]	(1) Participation in ketogenic diet intervention and epileptic seizures	Physician or parent observation (not stated which)	Physician or parent observation (not stated which)
Martinez-Brockman, 2023 (Eastern Caribbean Health Outcomes Research Network cohort study) [61]	Food insecurity and depressive symptoms	Survey—Latin American and Caribbean Food Security Scale	Patient Health Questionnaire (PHQ-2)
Mulenga, 2017 [70]	Food insecurity and suicide ideation	Survey—one question in WHO Global School-Based Student Health Survey	WHO Global School-Based Student Health Survey
Peltzer, 2015 ^c^ [63]	(1) Fruit intake and depressive symptoms; (2) Vegetable intake and depressive symptoms; (3) Fruit and vegetable intake and depressive symptoms	Survey—food frequency questionnaire	Centre for Epidemiologic Studies Depression Scale (CES-D)—10 item version
Peltzer, 2017 ^c^ [62]	(1) Eating breakfast daily and happiness; (2) Avoiding fat in diet and happiness; (3) Trying to eat more fibre and happiness; (4) Meals per day and happiness; (5) Snacks per day and happiness; (6) Fruit intake and happiness; (7) Veg per day and happiness; (8) Red meat intake and happiness	Survey—food frequency questionnaire	Subjective Happiness Scale (SHS)
Pengpid, 2020 ^c,f^ [66]	(1) Breakfast consumption and depressive symptoms; (2) Breakfast consumption and PTSD symptoms; (3) Breakfast consumption and happiness;	Survey—one question in researchers’ survey	Depression: Centres for Epidemiologic Studies Depression Scale (CES-D 10 items); PTSD: Breslau’s 7-item screener; Happiness: Subjective Happiness Scale (SHS)
Pengpid, 2023 ^f^ [67]	(1) Food insecurity and suicide ideation; (2) Food insecurity and suicide plan; (3) Food insecurity and suicide attempt; (4) Food insecurity and anxiety	Survey—one question in WHO Global School-Based Student Health Survey	WHO Global School-Based Student Health Survey
Racine, 2008 (Eastern Caribbean Child Vulnerability Study) [60]	(1) Food insecurity and presence of child with mental disability in HH; (2) Food insecurity and Child in HH with history of depression treatment	Survey—five questions in Child Vulnerability Survey	Child Vulnerability Survey
Rahbar, 2012 (Jamaican Autism study) ^d^ [71]	F&V: (1) [Yam, sweet potato or dasheen] intake and autism diagnosis; (2) [Carrot or pumpkin] intake and autism diagnosis; (3) Lettuce intake and autism diagnosis; (4) [Callaloo, broccoli, or pak choi] intake and autism diagnosis; (5) Cabbage intake and autism diagnosis; (6) String bean intake and autism diagnosis; (7) Tomato intake and autism diagnosis; (8) Ackee intake and autism diagnosis; (9) Avocado intake and autism diagnosis; SEAFOOD: (10) High seafood intake and autism diagnosis; (11) Seafood intake frequency and autism diagnosis; (12) Salt water fish intake and autism diagnosis; (13) Fresh water fish intake and autism diagnosis; (14) [Canned sardine, mackerel] fish intake and autism diagnosis; (15) Canned tuna intake and autism diagnosis; (16) Saltfish intake and autism diagnosis; (17) Shellfish intake and autism diagnosis; (18) Shrimp intake and autism diagnosis	Survey—food frequency questionnaire	CASES: (1) Autism Diagnostic Observation Schedule (ADOS), (2) Autism Diagnostic Interview-Revised (ADI-R); CONTROLS: Lifetime form of the Social Communication Questionnaire (SCQ)
Rahbar, 2013 (Jamaican Autism study) ^d^ [73]	(1) High seafood intake and autism diagnosis; (2) Seafood intake frequency and autism diagnosis; (3) Ate seafood and autism diagnosis; (4) Ate any fish and autism diagnosis; (5) Salt water fish intake and autism diagnosis; (6) Fresh water fish intake and autism diagnosis; (7) [Canned sardine, mackerel] fish intake and autism diagnosis; (8) Canned tuna intake and autism diagnosis; (9) Saltfish intake and autism diagnosis; (10) Shellfish intake and autism diagnosis; (11) Shrimp intake and autism diagnosis	Survey—food frequency questionnaire	CASES: (1) Autism Diagnostic Observation Schedule (ADOS), (2) Autism Diagnostic Interview-Revised (ADI-R); CONTROLS: Lifetime form of the Social Communication Questionnaire (SCQ)
Rahbar, 2014 (Jamaican Autism study) ^d^ [74]	F&V: (1) [Yam, sweet potato or dasheen] intake and autism diagnosis; (2) [Carrot or pumpkin] intake and autism diagnosis; (3) Lettuce intake and autism diagnosis; (4) [Callaloo, broccoli, or pak choi] intake and autism diagnosis; (5) Cabbage intake and autism diagnosis; (6) String bean intake and autism diagnosis; (7) Tomato intake and autism diagnosis; (8) Ackee intake and autism diagnosis; (9) Avocado intake and autism diagnosis; (10) Green banana intake and autism diagnosis; (11) Fried plantain intake and autism diagnosis; SEAFOOD: (12) High seafood intake and autism diagnosis; (13) Salt water fish intake and autism diagnosis; (14) Fresh water fish intake and autism diagnosis; (15) [Canned sardine, mackerel] fish intake and autism diagnosis; (16) Canned tuna intake and autism diagnosis; (17) Saltfish intake and autism diagnosis; (18) Shellfish intake and autism diagnosis; (19) Shrimp intake and autism diagnosis; GRAINS: (20) Rice intake and autism diagnosis; (21) Fried dumpling intake and autism diagnosis; (22) Boiled dumpling intake and autism diagnosis; (23) White bread intake and autism diagnosis; (24) Whole wheat bread intake and autism diagnosis; (25) [Cakes or buns] intake and autism diagnosis; (26) Porridge (corn or oatmeal) intake and autism diagnosis; (27) Cold breakfast cereal intake and autism diagnosis; ORGAN MEAT: (28) [Liver or kidney] intake and autism diagnosis.	Survey—food frequency questionnaire	CASES: (1) Autism Diagnostic Observation Schedule (ADOS), (2) Autism Diagnostic Interview-Revised (ADI-R); CONTROLS: Lifetime form of the Social Communication Questionnaire (SCQ)
Rahbar, 2014 (Jamaican Autism study) ^d^ [72]	F&V: (1) [Yam, sweet potato or dasheen] intake and autism diagnosis; (2) [Carrot or pumpkin] intake and autism diagnosis; (3) Lettuce intake and autism diagnosis; (4) [Callaloo, broccoli, or pak choi] intake and autism diagnosis; (5) Cabbage intake and autism diagnosis; (6) String bean intake and autism diagnosis; (7) Tomato intake and autism diagnosis; (8) Ackee intake and autism diagnosis; (9) Avocado intake and autism diagnosis; (10) Green banana intake and autism diagnosis; (11) Fried plantain intake and autism diagnosis; SEAFOOD: (12) High seafood intake and autism diagnosis; (13) Salt water fish intake and autism diagnosis; (14) Fresh water fish intake and autism diagnosis; (15) [Canned sardine, mackerel] fish intake and autism diagnosis; (16) Canned tuna intake and autism diagnosis; (17) Saltfish intake and autism diagnosis; (18) Shellfish intake and autism diagnosis; (19) Shrimp intake and autism diagnosis; GRAINS: (20) Rice intake and autism diagnosis; (21) Fried dumpling intake and autism diagnosis; (22) Boiled dumpling intake and autism diagnosis; (23) White bread intake and autism diagnosis; (24) Whole wheat bread intake and autism diagnosis; (25) [Cakes or buns] intake and autism diagnosis; (26) Porridge (corn or oatmeal) intake and autism diagnosis; (27) Cereal intake and autism diagnosis.	Survey—food frequency questionnaire	CASES: (1) Autism Diagnostic Observation Schedule (ADOS), (2) Autism Diagnostic Interview-Revised (ADI-R); CONTROLS: Lifetime form of the Social Communication Questionnaire (SCQ)
Rivera, 2023 [89]	“Various foods in diet” and “stress, anxiety”	Qualitative interview	Qualitative interview
Rocke, 2020 [90]	(1) Fish and meat diet pattern and depressive symptoms; (2) Fruit and vegetable diet pattern and depressive symptoms; (3) Western diet pattern and depressive symptoms; (4) Fish and meat diet pattern and perceived stress; (5) Fruit and vegetable diet pattern and perceived stress; (6) Western diet pattern and perceived stress.	Survey—food frequency questionnaire	Depression: 21-item Beck Depression Inventory (BDI); Stress: Cohen Perceived Stress Scale
Simeone, 2023 [91]	Difficulty obtaining food and post-partum depressive symptoms	Survey—one question in Pregnancy Risk Assessment Monitoring System (PRAMS), Disaster supplement	Pregnancy Risk Assessment Monitoring System (PRAMS), Disaster supplement
Siziya, 2017 [76]	Food security and suicide ideation	Survey—one question in WHO Global School-Based Student Health Survey	WHO Global School-Based Health Survey
Walker, 2006 (The Jamaican supplementation and stimulation study) ^e^ [92]	(1) Milk-based formula supplementation and depressive symptoms; (2) Milk-based formula supplementation and anxiety symptoms; (3) Milk-based formula supplementation and attention deficit symptoms; (4) Milk-based formula supplementation and oppositional behaviour symptoms	Diet not measured per se; but supplementation was given to the family each week for the infant and assumed to be used.	Depression: Short Mood and Feelings Questionnaire; Anxiety: Revised Children’s Manifest Anxiety Scale (RCMAS) Attention Deficit: Behaviour and Activities Checklist Oppositional Behaviour: Behaviour and Activities Checklist
Walker, 2011 (The Jamaican supplementation and stimulation study) ^e^ [93]	(1) Milk-based formula supplementation and depressive symptoms; (2) Milk-based formula supplementation and anxiety symptoms	Diet not measured per se; but supplementation was given to the family each week for the infant and assumed to be used.	Depression: Short Mood and Feelings Questionnaire; Anxiety: State-trait Anxiety Inventory

Footnotes: ^a^—Two records from the same study (10/66 Study); ^b^—Two records from the same study (Haiti Justice Sector Strengthening Project); ^c^—Three records from the same study (no study name); ^d^—Four records from the same study (Jamaican Autism Study); ^e^—Two records from the same study (Jamaican Supplementation and Stimulation Study); ^f^—Primary aim of record was to assess relationship between diet and mental health.

## Data Availability

No new data were created or analysed in this study. Data Sharing is not applicable to this article.

## Supplementary Materials

The following supporting information can be downloaded at: https://www.mdpi.com/article/10.3390/nu18010058/s1, Table S1: Database search terms (using syntax appropriate for PubMed); Table S2: List of references of 44 included records.

## Abbreviations

The following abbreviations are used in this manuscript:

EQ-5D-5L	EuroQol-5 Dimensions-5 Levels
LMIC	Low-and-Middle-Income Country
NCD	Non-communicable disease
SIDS	Small island developing state
WEIRD	Western, Educated, Industrialised, Rich and Democratic
WHO	World Health Organization
