# Supplementary material for: Diet and Mental Health Relationships in Caribbean Populations: A Scoping Review and Evidence Gap Map
Source: Nutrients. 2025 Dec 23;18(1):58. doi: 10.3390/nu18010058 (PMC12787968; doi:10.3390/nu18010058)
Supplement: Supplementary file 1 [file nutrients-18-00058-s001.zip › Table S1.pdf]

Table S1 – Database search terms (using syntax appropriate for PubMed)

Site: https://www.ncbi.nlm.nih.gov/pubmed

Limits: Title/Abstract; 01/Jan/2000 – 11/Feb/2024

**P**	*((Caribbean OR "West Indies" OR Leeward OR Windward OR Antilles OR Anguilla OR Antigua OR Aruba OR Barbuda OR Bahamas OR Barbados OR Barthelemy OR "St. Bartholomew" OR "Saint Bartholomew" OR Barts OR Belize OR Bermuda OR Bonaire OR Cayman OR Croix OR Cuba OR Curacao OR Dominica OR "Dominican Republic" OR Eustatius OR "Santo Domingo" OR "Saint Domingue" OR Grenada OR Grenadines OR Guadeloupe OR Guyana OR Haiti OR Hispaniola OR Jamaica OR "St. John" OR "Saint John" OR "St. Thomas" OR "Saint Thomas" OR "St. Vincent" OR "Saint Vincent" OR "St. Martin" OR "Saint Martin" OR "St. Maarten" OR "Saint Maarten" OR Martinique OR "St. Kitts" OR Kitts OR Nevis OR "St. Lucia" OR "Saint Lucia" OR Montserrat OR "Puerto Rico" OR Grenadines OR "Virgin Islands" OR Saba OR Suriname OR Trinidad OR Tobago OR Tortola)*
	AND
**I/E**	*(diet* OR eat OR nutrition* OR nutrient OR food OR "dietary intake" OR "diet diversity" OR "dietary diversity" OR protein OR "amino acid" OR sugar OR carbohydrate OR lipid OR "fatty acid" OR macronutrient OR micronutrient OR vitamin OR mineral OR antioxidant)*
	AND
**O**	*(mental OR neurologic* OR mood OR "affective disorder" OR psychiat* OR well-being OR wellbeing OR "quality of life" OR self-esteem OR mood OR sad OR sadness OR stress* OR dysthymi* OR dysphori* OR depress* OR anxiety OR bipolar OR schitzo* OR psychosis OR autism OR autistic OR dementia OR Alzheimer OR Parkinson OR trauma* OR compulsive OR "oppositional defiant" OR "conduct disorder" OR "attention deficit" OR ADHD OR "dissociation disorder" OR "dissociative disorder" OR "disruptive mood dysregulation disorder" OR DMDD OR anorexia OR bulimia OR binge OR post-traumatic OR paranoia OR phobia OR panic OR suicid* OR parasuicid* OR self-harm OR addict* OR insomnia))*
	**OR**
**P**	*((Caribbean OR "West Indies" OR Leeward OR Windward OR Antilles OR Anguilla OR Antigua OR Aruba OR Barbuda OR Bahamas OR Barbados OR Barthelemy OR "St. Bartholomew" OR "Saint Bartholomew" OR Barts OR Belize OR Bermuda OR Bonaire OR Cayman OR Croix OR Cuba OR Curacao OR Dominica OR "Dominican Republic" OR Eustatius OR "Santo Domingo" OR "Saint Domingue" OR Grenada OR Grenadines OR Guadeloupe OR Guyana OR Haiti OR Hispaniola OR Jamaica OR "St. John" OR "Saint John" OR "St. Thomas" OR "Saint Thomas" OR "St. Vincent" OR "Saint Vincent" OR "St. Martin" OR "Saint Martin" OR "St. Maarten" OR "Saint Maarten" OR Martinique OR "St. Kitts" OR Kitts OR Nevis OR "St. Lucia" OR "Saint Lucia" OR Montserrat OR "Puerto Rico" OR Grenadines OR "Virgin Islands" OR Saba OR Suriname OR Trinidad OR Tobago OR Tortola)*
	AND
**(I/E/O)**	*("nutritional psychiatry" OR "nutrition psychiatry" OR "nutritional psychology" OR "nutrition psychology" OR psychodietetics OR nutripsychiatry))*
