# Supplementary material for: Diet and Mental Health Relationships in Caribbean Populations: A Scoping Review and Evidence Gap Map
Source: Nutrients. 2025 Dec 23;18(1):58. doi: 10.3390/nu18010058 (PMC12787968; doi:10.3390/nu18010058)
Supplement: Supplementary file 1 [file nutrients-18-00058-s001.zip › Table S2.pdf]

Table S2 – List of references of 44 included records.

Diet-Mental Health Relationship Records (n=35)

1. Albanese E, Dangour AD, Uauy R, Acosta D, Guerra M, Guerra SSG, et al. Dietary fish and meat intake and dementia in Latin America, China, and India: a 10/66 Dementia Research Group population-based study. Am J Clin Nutr. 2009 Aug;90(2):392–400.
2. Albanese E, Lombardo FL, Dangour AD, Guerra M, Acosta D, Huang Y, et al. No association between fish intake and depression in over 15,000 older adults from seven low and middle income countries--the 10/66 study. PloS One. 2012;7(6):e38879.
3. Augustus E, Granderson I, Rocke KD. The Impact of a Ketogenic Dietary Intervention on the Quality of Life of Stage II and III Cancer Patients: A Randomized Controlled Trial in the Caribbean. Nutr Cancer. 2021 Aug 18;73(9):1590–600.
4. Benites-Zapata VA, Urrunaga-Pastor D, Solorzano-Vargas ML, Herrera-Añazco P, Uyen-Cateriano A, Bendezu-Quispe G, et al. Prevalence and factors associated with food insecurity in Latin America and the Caribbean during the first wave of the COVID-19 pandemic. Heliyon. 2021 Oct;7(10):e08091.
5. Brewis A, Wutich A, Galvin M, Lachaud J. Localizing syndemics: A comparative study of hunger, stigma, suffering, and crime exposure in three Haitian communities. Soc Sci Med. 2022 Feb 1;295:113031.
6. Celeste-Villalvir A, Then-Paulino A, Armenta G, Jimenez-Paulino G, Palar K, Wallace DD, et al. Exploring feasibility and acceptability of an integrated urban gardens and peer nutritional counselling intervention for people with HIV in the Dominican Republic. Public Health Nutr. 2023 Dec;26(12):3134–46.
7. Cleret de Langavant L, Roze E, Petit A, Tressières B, Gharbi-Meliani A, Chaumont H, et al. Annonaceae Consumption Worsens Disease Severity and Cognitive Deficits in Degenerative Parkinsonism. Mov Disord Off J Mov Disord Soc. 2022 Dec;37(12):2355–66.
8. DaSantos A, Goddard C, Ragoobirsingh D. Self-care adherence and affective disorders in Barbadian adults with type 2 diabetes. AIMS Public Health. 2022;9(1):62–72.
9. Ertl MM, Trapp SK, Alzueta E, Baker FC, Perrin PB, Caffarra S, et al. Trauma-Related Distress During the COVID-19 Pandemic In 59 Countries. Couns Psychol. 2022 Apr 1;50(3):306–34.
10. Fabián C, Pagán I, Ríos JL, Betancourt J, Cruz SY, González AM, et al. Dietary patterns and their association with sociodemographic characteristics and perceived academic stress of college students in Puerto Rico. P R Health Sci J. 2013 Mar;32(1):36–43.
11. Jeffers NK, Wilson D, Tappis H, Bertrand D, Veenema T, Glass N. Experiences of pregnant women exposed to Hurricanes Irma and Maria in the US Virgin Islands: a qualitative study. BMC Pregnancy Childbirth. 2022 Dec 17;22(1):947.
12. Jones AD. Food Insecurity and Mental Health Status: A Global Analysis of 149 Countries. Am J Prev Med. 2017 Aug;53(2):264–73.
13. Koyanagi A, Stubbs B, Oh H, Veronese N, Smith L, Haro JM, et al. Food insecurity (hunger) and suicide attempts among 179,771 adolescents attending school from 9 high-income, 31 middle-income, and 4 low-income countries: A cross-sectional study. J Affect Disord. 2019 Apr 1;248:91–8.
14. Kwangu M, Siziya S, Mulenga D, Mazyanga M, Njunju E. Correlates of suicidal ideation among school-going adolescents in Bahamas. Int Public Health J. 2017;9(14):393–9.
15. Lachaud J, Hruschka DJ, Kaiser BN, Brewis A. Agricultural wealth better predicts mental wellbeing than market wealth among highly vulnerable households in Haiti: Evidence for the benefits of a multidimensional approach to poverty. Am J Hum Biol Off J Hum Biol Counc. 2020 Mar;32(2):e23328.
16. LaMonaca K, Desai M, May JP, Lyon E, Altice FL. Prisoner health status at three rural Haitian prisons. Int J Prison Health. 2018 Sep 10;14(3):197–209.
17. López-Cepero A, O'Neill J, Tamez M, Falcón LM, Tucker KL, Rodríguez-Orengo JF, et al. Associations Between Perceived Stress and Dietary Intake in Adults in Puerto Rico. J Acad Nutr Diet. 2021 Apr 1;121(4):762–9.
18. Marcos Plasencia LM, Rojas Massipe E. Presentación de un caso de aplicación de la dieta cetogénica en la epilepsia refractaria. Rev Cuba Pediatría. 2007 Dec;79(4):0–0.
19. Martinez-Brockman JL, Hromi-Fiedler A, Galusha D, Oladele C, Acosta L, Adams OP, et al. Risk factors for household food insecurity in the Eastern Caribbean Health Outcomes Research Network cohort study. Front Public Health. 2023;11:1269857.
20. Mulenga D, Siziya S, Mazaba ML, Kwangu M, Njunju EM. Correlates of suicidal ideation among in-school adolescents in Trinidad and Tobago. Int Public Health J. 2017;9(4):437.
21. Peltzer K, Pengpid S. Correlates of healthy fruit and vegetable diet in students in low, middle and high income countries. Int J Public Health. 2015 Jan;60(1):79–90.
22. Peltzer K, Pengpid S, Sodi T, Mantilla Toloza SC. Happiness and health behaviours among university students from 24 low, middle and high income countries. J Psychol Afr. 2017 Feb 27;27(1):61–8.

23. Pengpid S, Peltzer K. Skipping Breakfast and Its Association with Health Risk Behaviour and Mental Health Among University Students in 28 Countries. Diabetes Metab Syndr Obes Targets Ther. 2020;13:2889–97.
24. Pengpid S, Peltzer K. Food insecurity is associated with multiple psychological and behavioural problems among adolescents in five Caribbean countries. Psychol Health Med. 2023 Feb;28(2):291–304.
25. Racine EF, Jemison K, Huber LR, Arif AA. The well-being of children in food-insecure households: results from The Eastern Caribbean Child Vulnerability Study 2005. Public Health Nutr. 2009 Sep;12(9):1443–50.
26. Rahbar MH, Samms-Vaughan M, Ardjomand-Hessabi M, Loveland KA, Dickerson AS, Chen Z, et al. The role of drinking water sources, consumption of vegetables and seafood in relation to blood arsenic concentrations of Jamaican children with and without Autism Spectrum Disorders. Sci Total Environ. 2012 Sep 1;433C:362–70.
27. Rahbar MH, Samms-Vaughan M, Dickerson AS, Loveland KA, Ardjomand-Hessabi M, Bressler J, et al. Blood manganese concentrations in Jamaican children with and without autism spectrum disorders. Environ Health. 2014 Aug 23;13:69.
28. Rahbar MH, Samms-Vaughan M, Dickerson AS, Loveland KA, Ardjomand-Hessabi M, Bressler J, et al. Role of fruits, grains, and seafood consumption in blood cadmium concentrations of Jamaican children with and without Autism Spectrum Disorder. Res Autism Spectr Disord. 2014 Sep 1;8(9):1134–45.
29. Rahbar MH, Samms-Vaughan M, Loveland KA, Ardjomand-Hessabi M, Chen Z, Bressler J, et al. Seafood Consumption and Blood Mercury Concentrations in Jamaican Children With and Without Autism Spectrum Disorders. Neurotox Res. 2013 Jan 1;23(1):22–38.
30. Rivera I. Examining Dominican folk knowledge and practices used as self-care during crises in the Dominican Republic [Internet]. [Chicago]: The Chicago School of Professional Psychology; 2023 [cited 2025 Jul 14]. Available from: https://www.proquest.com/openview/1c53b8a1f8dae052b6c0cef250a821e3/1?pq-origsite=gscholar&cbl=18750&diss=y
31. Rocke K, Roopchand X. Predictors for depression and perceived stress among a small island developing state university population. Psychol Health Med. 2021 Oct;26(9):1108–17.
32. Simeone RM, House LD, Salvesen von Essen B, Kortsmit K, Hernandez Virella W, Vargas Bernal MI, et al. Pregnant Women's Experiences During and After Hurricanes Irma and Maria, Pregnancy Risk Assessment Monitoring System, Puerto Rico, 2018. Public Health Rep Wash DC 1974. 2023;138(6):916–24.
33. Siziya S, Njunju EM, Kwangu M, Mulenga D, Mazaba-Liwewe M. Suicidal ideation in Jamaica. In: Suicide: A Global View on Suicidal Ideation among Adolescents. 2017. p. 85–98.
34. Walker SP, Chang SM, Vera-Hernández M, Grantham-McGregor S. Early childhood stimulation benefits adult competence and reduces violent behavior. Pediatrics. 2011 May;127(5):849–57.
35. Walker SP, Chang SM, Powell CA, Simonoff E, Grantham-McGregor SM. Effects of psychosocial stimulation and dietary supplementation in early childhood on psychosocial functioning in late adolescence: follow-up of randomised controlled trial. BMJ. 2006 Sep 2;333(7566):472.

Eating Disorder Records (n=9)

36. Bhugra D, Mastrogianni A, Maharajh H, Harvey S. Prevalence of bulimic behaviours and eating attitudes in schoolgirls from Trinidad and Barbados. Transcult Psychiatry. 2003 Sep;40(3):409–28.
37. Hoek HW, van Harten PN, Hermans KME, Katzman MA, Matroos GE, Susser ES. The incidence of anorexia nervosa on Curaçao. Am J Psychiatry. 2005 Apr;162(4):748–52.
38. Katzman MA, Hermans KME, Hoeken DV, Hoek HW. Not Your "Typical Island Woman": Anorexia Nervosa is Reported Only in Subcultures in Curaçao. Cult Med Psychiatry. 2004 Dec 1;28(4):463–92.
39. McGuire MT, Story M, Neumark-Sztainer D, Halcon L, Campbell-Forrester S, Blum RW. Prevalence and correlates of weight-control behaviors among Caribbean adolescent students. J Adolesc Health Off Publ Soc Adolesc Med. 2002 Aug;31(2):208–11.
40. Nichols SD, Dookeran SS, Ragbir KK, Dalrymple N. Body image perception and the risk of unhealthy behaviours among university students. West Indian Med J. 2009 Nov;58(5):465–71.
41. Ramberan K, Austin M, Nichols S. Ethnicity, body image perception and weight-related behaviour among adolescent Females attending secondary school in Trinidad. West Indian Med J. 2006 Dec;55(6):388–93.
42. Reyes-Rodríguez ML, Franko DL, Matos-Lamourt A, Bulik CM, Von Holle A, Cámara-Fuentes LR, et al. Eating Disorder Symptomatology: Prevalence among Latino College Freshmen Students. J Clin Psychol. 2010 Jun;66(6):666–79.
43. Reyes-Rodríguez ML, Sala M, Von Holle A, Unikel C, Bulik CM, Cámara-Fuentes L, et al. A Description of Disordered Eating Behaviors in Latino Males. J Am Coll Health. 2011 Jan 21;59(4):266–72.

44. White VO, Gardner JM. Presence of anorexia nervosa and bulimia nervosa in Jamaica. West Indian Med J. 2002 Mar;51(1):32–4.
